# Powdered plant beverages obtained by spray-drying without carrier addition-physicochemical and chemometric studies

**DOI:** 10.1038/s41598-024-54978-x

**Published:** 2024-02-23

**Authors:** Katarzyna Samborska, Iwona Budziak-Wieczorek, Arkadiusz Matwijczuk, Dorota Witrowa-Rajchert, Mariusz Gagoś, Bożena Gładyszewska, Dariusz Karcz, Katarzyna Rybak, Maciej Jaskulski, Alicja Barańska, Aleksandra Jedlińska

**Affiliations:** 1 Department of Food Engineering and Process Management, Institute of Food Sciences, Warsaw University of Life Sciences WULS-SGGW, Nowoursynowska 159C, 02-776 Warsaw, Poland; 2 Department of Chemistry, Faculty of Life Sciences and Biotechnology, University of Life Sciences in Lublin, Akademicka 15, 20-950 Lublin, Poland; 3 Department of Biophysics, Faculty of Environmental Biology, University of Life Sciences in Lublin, Akademicka 13, 20-950 Lublin, Poland; 4 Department of Biochemistry and Molecular Biology, Medical University of Lublin, 20-093 Lublin, Poland; 5 Department of Environmental Engineering, Faculty of Process and Environmental Engineering, Lodz University of Technology, Wólczańska 213, 93-005 Łódź, Poland; 6 Department of Chemical Technology and Environmental Analytics, Krakow University of Technology, 31-155 Krakow, Poland; 7 ECOTECH-COMPLEX—Analytical and Programme Centre for Advanced Environmentally-Friendly Technologies, Maria Curie-Sklodowska University, Głęboka 39, 20-033 Lublin, Poland

**Keywords:** Infrared spectroscopy, Sustainability, Nutrition, Biotechnology, Materials science

## Abstract

Plant-based beverages (PBs) are currently gaining interest among consumers who are seeking alternative sustainable options to traditional dairy drinks. The study aimed to obtain powdered plant beverages without the addition of carriers by spray drying method to implement them in the future as an alternative to the liquid form of dairy drinks. Some of the most well-known commercial beverages sources like soy, almond, rice and oat were analyzed in this work. The effect of different treatments (concentration, addition of oat fiber) and two approaches od spray drying (conventional high temperature spray drying—SD, and dehumidified air spray drying at low temperature—DASD) were presented. Moreover, moisture content, water activity, particle morphology and size of obtained powders were analyzed. It was possible to obtain PBs without the addition of carriers, although the drying yield of four basic beverages was low (16.1–37.4%). The treatments and change in spray drying approach enhanced the drying yield, especially for the concentrated beverage dried using DASD (59.2%). Additionally, Fourier Transform Infrared (FTIR) spectroscopy was applied to evaluate the differences in chemical composition of powdered PBs. FTIR analysis revealed differences in the range of the absorption frequency of amide I, amide II (1700–1500 cm^−1^) and carbohydrate region (1200–900 cm^−1^). Principal component analysis (PCA) was carried out to study the relationship between spray dried plant beverages samples based on the fingerprint region of FTIR spectra, as well as the physical characteristics. Additionally, hierarchical cluster analysis (HCA) was employed to explore the clustering of the powders.

## Introduction

The consumption of plant-based beverages (PBs) continues to increase, especially in developing countries, due to different reasons: the growth of bovine milk protein allergy and lactose intolerance among consumers; the increasing popularity of vegetarian and vegan diets; growing awareness of the impact of animal production on the environment^1,2^. According to Silva et al^3^., about 75% of the world’s population has symptoms of lactose intolerance caused by the deficiency of lactase enzyme and poor digestion of lactose.

PBs are water-soluble extracts of legumes, oilseeds, cereals or pseudocereals that resemble bovine milk in appearance^3^. Soy, almond, rice, and coconut milk are widely produced and consumed in a liquid form, while other PBs such as oat or wheat also draw attention. Different types of commercial PBs available in the market differ in the amount of macro and micronutrients and the presence of bioactive compounds. Additionally, these products very often contain some additives, including those added for fortification (vitamins, extracts with phytochemicals), taste (sweeteners) or structure (thickeners). As for bioactive compounds, for example, soy-based beverage contains phytochemicals like isoflavones and saponins; almond-based beverage contains *α*-tocopherol and arabinose; oat-based beverage contains *β*-glucan; rice-based beverage contains *α*-tocopherol and *γ*-oryzanol^3^.

Powdered form of PBs is an alternative to liquid form, and similarly as powdered milk has prolonged shelf life, reduced mass and volume, offer easy and convenient handling. Spray drying is the most widely applied method for production of food powders from liquid raw materials^4^. It is well known that the application of drying carriers (among them the most popular is maltodextrin) is a method to facilitate drying process and improve the characteristics of powders. For example, Yulistiani et al^5^. presented that coconut milk powder without carrier had low yield value, clumps, yellowish white colour, and sticked to the drying equipment, while the addition of maltodextrin facilitated the drying process and improved the properties of the coconut milk powder. Many different types of PBs were spray dried to investigate the impact of drying conditions and/or the addition of carriers on powder yield and properties: almond enriched with probiotics^6^, cereal-based fermented beverage Boza^7^, soy^8^, coconut beverage^9^. Usually maltodextrin has been applied as the carrier–ingredient improving the process performance. However, nowadays consumers are more aware and are expecting products with “clean label” features. Thus, there is a strong need to verify the possibility to spray dry PB without the carrier addition, and to find any other method/pretreatment to enhance drying yield and improve powder properties. Padma et al^10^. spray dried rice beverage without carrier at inlet air temperature 120–160 °C, but the drying yield was not specified.

Dehumidified air spray drying (DASD) at low temperature is a novel approach in spray drying process, which applies inlet air of reduced humidity to supply additional driving force for evaporation^11^. It allows to decrease air temperature to avoid stickiness. It is especially devoted for spray drying of sugar-rich materials, which are very vulnerable for stickiness due to low glass transition of low molecular weight sugars. The assumption of this work was that it can be similarly applied for PBs spray drying as a method to improve the process yield and eliminated the carrier addition.

The combination of Fourier-Transform InfraRed spectroscopy (FTIR) with multivariate analysis such as Principal Component Analysis (PCA) and Hierarchical Cluster Analysis (HCA) are the most frequent methods used in classification, determination of authenticity and adulteration in foods^12–14^ and as an alternative technology for quality and safety control in dairy industry^14,15^. However, in the literature, there are few examples of the application of FTIR spectroscopy coupled with chemometric techniques for spray-dried products^16–18^. Therefore, in our study, we used FTIR spectroscopy to determine the “fingerprint” of powdered PBs, and further combined with PCA and HCA for search the similarity and structural patterns among powdered plant beverages with different botanical origins.

Nowadays, healthy nutrition trends in the food industry promote the development of spray dryied products to obtain powders without unnecessary additives. Therefore, this study primarily aimed to obtain powdered plant beverages without the addition of carriers, and characterize the performance of drying and the physicochemical properties of powders. This work presents results of spray drying of four basic types of commercial PBs as well as spray drying of oat beverage to improve yield by pretreatments and/or modification in drying process. Moreover, FTIR spectroscopy coupled with chemometric tools (PCA and HCA) was applied as an alternative analysis to study relationships among the spectra and various physical properties and drying yield.

## Materials and methods

### Materials

Four types of commercial PBs were used as raw material for spray drying: soy beverage (Alpro, Gent, Belgium; SOY), almond beverage (Scotti, Pavia, Italy; ALMOND), rice beverage (Bio Zen—PolBioEko, Lublin, Polska; RICE), oat beverage Inka (Grana, Skawina, Poland; OAT). Oat fiber (Młyn Oliwski, Gdańsk, Poland) was applied as additional component potentially improving nutritional value of oat beverage. The ingredients and nutritional value of PBs are presented in Table 1. The fat, carbohydrates and proteins content were summarized and presented as solids content, for further calculation of the share of sugars in carbohydrates and the share of protein in solids, as these two factors could potentially affect the progress of spray drying (as described in the further parts of the manuscript).

**Table 1:** The ingredients and nutritional value of commercial plant beverages, with additional calculation of solids content, the share of sugars in carbohydrates and the share of protein in solids.

Sample/producer	Ingredients	Nutritional value in 100 mL [g]			Solids [%. w/w]	Sugars share in carbohydrates [%, w/w]	Protein share in solids [%, w/w]
		Fat (incl. saturated)	Carbohydrates (incl. sugars) (fiber)	Protein			
SOY/Alpro	Soy base (water, dehulled soybean (8,7%)), apple extract, regulatory kwasowości (fosforan potasu), calcium, sea salt (0.1%), stabilizer (gellan gum), vitamins (B2, B12, D2)	1,9 (0.3)	2,7 (2.5) (0.6)	3,3	7,9	92,6	41,8
ALMOND/Scotti	Water, almonds (5%) stabilizer (gellan gum), salt (0.1%)	2,4 (0.2)	1 (0.2) (0.5)	0,9	4,3	20	20,9
RICE/Bio Zen	Water, rice (14%), sunflower oil, sea salt (0.1%)	1 (0.1)	11 (5.0) (0.5)	0,5	12,5	45,5	4
OAT/INKA	Water, oats 10%, sunflower oil, calcium carbonate, sea salt (0.1%), vitamins: E, B6, A, folic acid, D, B12	1,1 (0.2)	7,6 (4.4) (0.5)	0,7	9,4	57,9	7,4

#### Experimental variants and spray drying

The experimental variants are presented in Table 2. In total nine variants of PBs were spray dried, including four basic types of commercial PBs, and additionally five variants of oat PB with additional treatments (concentration to double solids content, addition of oat fiber) or different spray drying approach (as described below). Oat beverage was chosen for the second part of work because among four basic PBs it had the lowest yield of drying, and this part aimed at the improvement of yield by the modification of beverage and/or drying process.

**Table 2:** Experimental variants of spray dried plant-based bevarages.

Variant name	Material	Treatment	Spray drying conditions*
SOY	Commercial soy beverage	–	180 °C/90 °C
ALMOND	Commercial almond beverage	–	180 °C/90 °C
RICE	Commercial rice beverage	–	180 °C/90 °C
OAT	Commercial oat beverage	–	180 °C/90 °C
CONC		Concentrated 2x	180 °C/90 °C
DASD**		–	90 °C/60 °C
CONC-DASD		Concentrated 2x	90 °C/60 °C
FIB-DASD		Addition of oat fiber	90 °C/60 °C
FIB-CONC-DASD		Concentrated 2×, addition of oat fiber	90 °C/60 °C

* inlet/outlet air temperature. Outlet air temperature is regarded as actual temperature of the material during the process.

** DASD – dehumidified air spray drying—the process in which the drying temperature can be reduced due to reduction of air humidity. Low air humidity provides additional driving force for water evaporation and the drying is possible even at low temperature.

Spray drying was performed using a NIRO Minor (GEA) pilot-scale dryer in a co-current flow. 500 g of the beverage was fed to the rotating disc with the feed ratio speed of 0.3 ml/s at inlet/outlet air temperature of 180 °C/90 °C (SD) and 90 °C/60 °C (DASD)^19,20^. The compressed air pressure was 4.5 bar, the rotational speed of disc was 26,000 rpm. After drying the powder was collected also from internal walls of the drier, by wiping with a stream of air to the jar placed under the cyclone. The powder was weighed and the yield (Y) was calculated and expressed in percentage of solids recovered.

#### Particle morphology—SEM analysis

The morphology of the powder particles was described based on photos taken with the XL scanning electron microscope (PHENOM) at a magnification of 500 × at an accelerating voltage of 5 kV. Before the photos were taken, the samples were covered with a layer of gold using Sputter Coater 108 auto (Cressington)^21^.

#### Particle size measurement

Measurements were made by laser diffraction using an 1190 device (CILAS) in liquid (ethanol) dispersion at maximum obscuration 10%^22^. The particle size distribution and the cumulative particle size distribution were derived graphically. The median diameter *D*_*50*_ was also presented, while parameters *D*_*10*_ and *D*_*90*_ of volumetric fractions (values of the dimension below which 10 and 90% of all particles occur, respectively) were used to calculated the polydispersity index (PDI) based on the formula: PDI = (D_90_-D_10_)/D_50_.

#### Moisture content and water activity

The moisture content (MC) in the powders was determined by the oven method. About 0.5 g of samples was weighed on an analytical balance (accuracy of 0.0001 g) and then dried at the temperature of 105 °C during 4 h. The water activity (*a*_*w*_) of the powders was determined using the HygroLab C1 (Rotronic) device. The measurements were made at the temperature of 25 °C in duplicate^23^.

#### FTIR

Measurements of infrared spectra were conducted with the use of a 670-IR spectrometer (Agilent, Santa Clara, CA, USA)^24^ at 23 °C. An ATR (Attenuated Total Reflection) attachment was used in the form of a ZnSe crystal with adequate geometry (truncated at 45°) to ensure 20-fold internal reflection of the absorbed beam. The powders were pressed onto the crystal surface. During the measurement, 16 scans were registered and subsequently the programme averaged the results for all spectra. Prior to the measurement, the ZnSe crystal was cleaned using pure organic solvents by Sigma-Aldrich (Darmstadt, Germany). Spectral measurements were recorded in the region from 400 to 4000 cm^−1^ at the resolution of 1 cm^−1^.

### Statistical analysis

#### Analysis of variance (ANOVA) 

All measurement were done in triplicate and the results were expressed as mean ± standard deviations. One-way analysis of variance (ANOVA) and Tukey’s test (*p* < 0.05) were used to establish the significance of differences among the mean values. The data were analysed using STATISTICA 13.3 software (Statsoft, Tulsa, OK, USA).

#### Multivariate analysis 

To investigate the differences in the yield coupled with physical properties, and separately in FTIR spectra of various powdered PBs unsupervised multivariate methods were used, including PCA and HCA. Chemometrics analysis (PCA and HCA) were performed using the Statistica 13 software (TIBCO Software Inc. PaloAlto, CA,USA) and and OriginPro (OriginLab Corporation, Northampton, MA, USA). FTIR spectra before PCA and HCA were preprocessed using Grams/AI 8.0 software (Thermo Scientific, Waltham, MA, USA) and OriginPro (OriginLab Corporation, Northampton, MA, USA). Before the chemometric analysis the spectra were subjected to pretreatment using multi-point baseline correction, Y offset correlation, mean center and/or second-order derivative using Savitzky-Golay smoothing algorithm with 20 points.

**PCA.** PCA is one of the most popular multivariate method to reduce a large set of correlated variables to uncorrelated latent variables called principal components (PC). Each of PC is a particular linear combination of the original quality characteristics and still explains all the variance in the matrix of the original variables^25,26^. PCs are orthogonal to each other and usually the first component explains the largest possible variances. The goal of PCA is to obtain multiple-variable system to detect data structure, enable the classification between samples, and determine general relationship among data. The PCA is an exploratory technique, which is based on the following expression:

1 $${\text{X}}\, = \,{\text{TP}}^{{\text{T}}} \, + \,{\text{E}}$$

where X is the data matrix to be analysed, T is called score matrix, P the loading matrix, and E the residual.

**HCA.** Hierarchical clustering analysis is one of the exploratory methods used to classify items into clusters (groups) based on distance that is calculated from distance matrix and the similarity between them^27^. Graphical representation of results is a tree graph called a dendrogram. HCA is based on finding the smallest distances between items (such as spectroscopic spectra) and the measure of dissimilarity between sets of observations. In hierarchical clustering the appropriate method of measuring the distance between pairs of observations and linkage criteria should be used. As it is distinct from other clustering algorithms, in this case it is not necessary to predeterminate the number of created clusters. In HCA, Pearson correlation and Euclidean distance between the pairs of samples were used as a distance measure and the average linkage and complete linkage criteria were used as an agglomeration method.

### Results and discussion

Each drying experiment performed was successful and allowed for isolation of the powder. However, the process performance and physical properties od powders were dependent on the type of PB and the applied modification of the beverage or of the drying itself. Detailed impacts are described below.

#### Particle morphology and size

**Figure 1:**
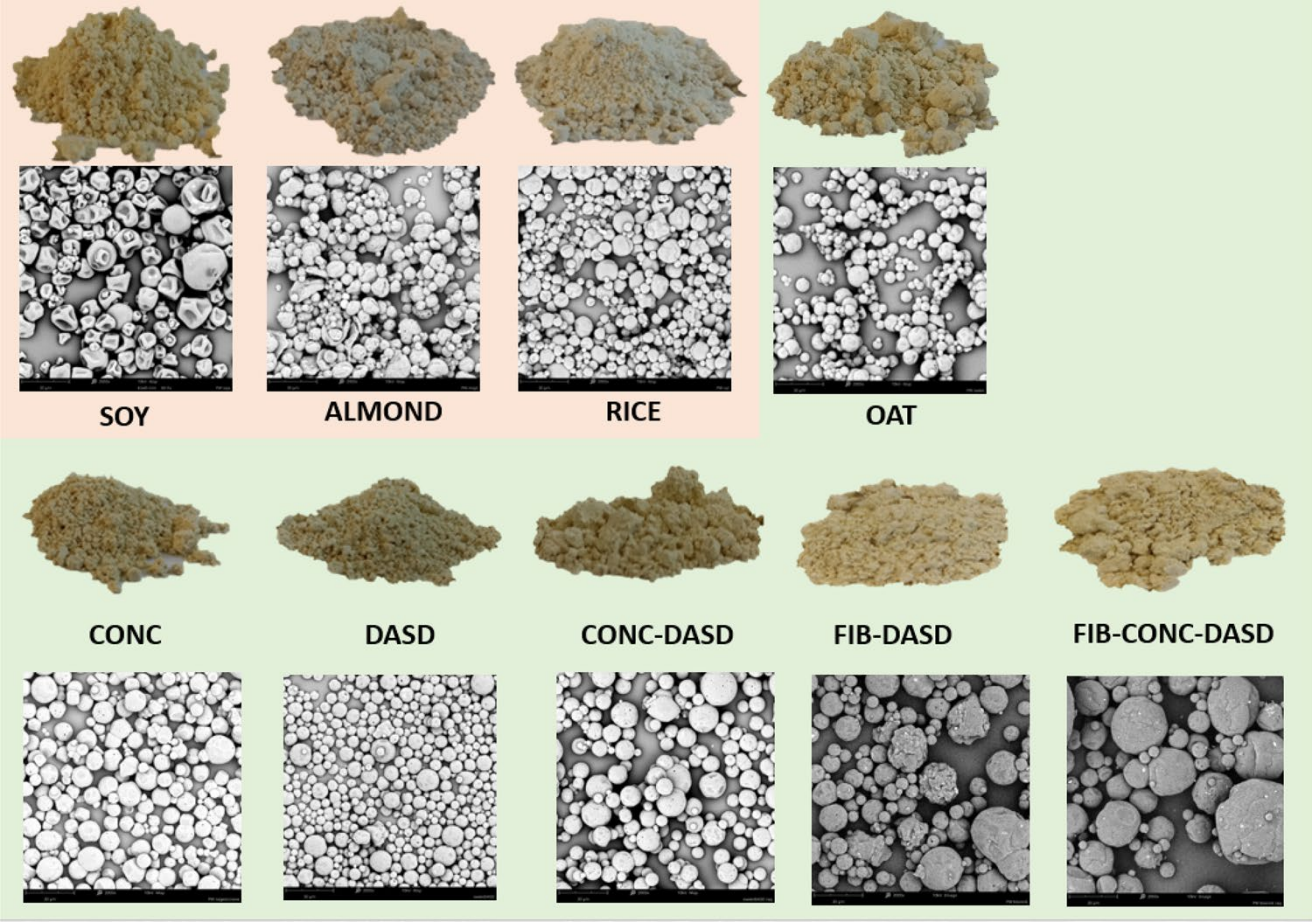
The appearance of powders and particles and SEM images (mag. 500 ×), (experimental variants marked as in Table 2).

**Figure 2:**
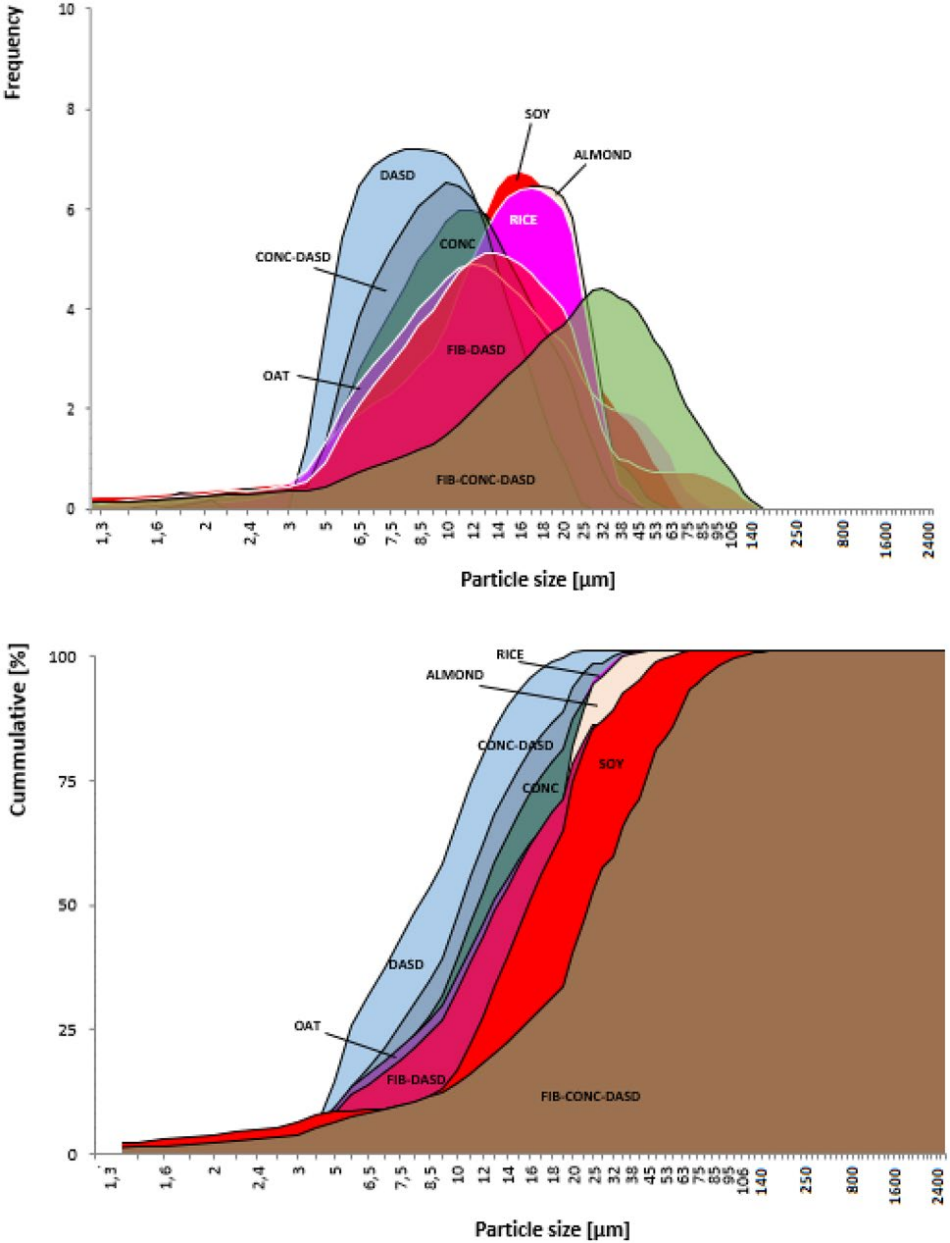
Particle size distribution and cumulative particle size distribution of powdered plant-based beverages (experimental variants marked as in Table 2).

All powders were fully flowable and had very similar physical overall appearance (Fig. 1). However, the differences could be observed in the morphology of particles presented in pictures taken by scanning electron microscope (Fig. 1). Among four basic powdered PBs the sample of SOY beverage had different morphology of particles. They were not so regular and round as for other types of beverages. The surfaces of SOY particles were wrinkled, contained convexities, and the shape was similar to golf ball. Very similar morphology of spray dried soy beverage was presented before by Jinapong et al^28^. On the contrary, other three basic types of powders had regular round particles. Such differences could be potentially the effect of the differences in chemical composition between these four samples^29^. SOY beverage contained the highest amount of protein (Table 1), which was 41.8% (w/w) of total solids. Moreover, the share of sugars in carbohydrates was also much different (higher) in SOY than in the rest of basic beverages – it was almost close to 100% (92.6%, w/w). Such differences could result in different particle morphology, because the chemical composition affects the formation of hard crust on the surface of droplets during water evaporation, which in turn affect the ability of formed particles to shrink or to stay in a regular round shape. Usually the presence of compounds of high glass transition temperature and film-forming properties (as polysaccharides) causes during evaporation the fast formation of hard crust which prevents the shrinkage and corrugation. It is also known that proteins are also characterized by film-forming ability^30^, but such protein-rich film is more elastic than formed by polysaccharides, and particles can shrink^30^. As presented by Elversson & Millqvist-Fureby^31^ spray dried particles containing proteins display a characteristic raisin-like corrugated morphology due to the adsorption of protein at the air/liquid interface of the droplets in the spray. Similar effect was presented by Brech et al^30^. during spray drying of amorphous lactose–the addition of long flexible molecules such as caseins, which increase the elasticity of the particle wall, resulted in folded particle surfaces. Particle–wall elasticity allows more expansion of internal-vacuole-containing particulates in the hotter regions of the spray dryer and deflation of these particulates and folding of their surfaces in cooler regions of the drier. Thus, it is the interplay between saccharides and proteins which affects the formation of particle morphology. In fact, SOY beverage, characterized by high share of protein, had also such corrugated and wrinkled morphology. Another reason why increased amount of protein can alter the morphology is connected with the differences in drying rate. Protein-rich film on the surface of droplets decreases the evaporation rate^21^, thus the external crust is not formed so quickly and the particle can shrink. Such phenomenon was observed i.e. by Elversson & Millqvist-Fureby^31^ who presented that the addition of bovine serum albumin changed the morphology from smooth to highly wrinkled. The above-described relationships however did not affect significantly the particle size measured by laser diffraction, because the wrinkling happened within similar ranges of diameters of four basic types of powdered PBs (Fig. 2, Table 3).

**Table 3:** Yield (*Y*), water activity (*a*_*w*_), moisture content (MC), median particle size (*D*_*50*_ ) and polydispersicity index (PDI) of obtained powders.

Sample	*Y* [%]	*a*_*w*_	MC [%]	D_50_ [μm]	PDI
SOY	32.0 ± 1.1 c	0.153 ± 0.001 b	4.5 ± 0.2 d	15.8 ± 0.2 b	1.6 ± 0.1 a
ALMOND	24.7 ± 1.5 b	0.179 ± 0.002 c	3.6 ± 0.1 cd	14.4 ± 0.6 b	1.3 ± 0.1 a
RICE	37.4 ± 0.5 cd	0.079 ± 0.001 a	2.6 ± 0.1 b	13.6 ± 0.6 b	1.3 ± 0.1 a
OAT	16.1 ± 0.7 a	0.156 ± 0.003 b	2.5 ± 0.2 b	12.3 ± 0.5 b	2.4 ± 0.2 b
CONC		0.080 ± 0.003 a	1.3 ± 0.1 a	11.7 ± 0.3 b	1.5 ± 0.2 a
DASD	44.6 ± 0.7 d	0.150 ± 0.001 b	3.5 ± 0.2 c	8.2 ± 0.2 a	1.2 ± 0.2 a
CONC-DASD	59.2 ± 3.6 e	0.157 ± 0.002 b	3.2 ± 0.1 c	10.3 ± 0.1 b	1.4 ± 0.1 a
FIB-DASD	32.5 ± 5.0 c	0.259 ± 0.001 d	5.2 ± 0.3 e	13.3 ± 0.1 b	2.2 ± 0.3 b
FIB-CONC-DASD	37.3 ± 0.6 cd	0.181 ± 0.001 c	3.6 ± 0.1 c	26.8 ± 1.1 c	2.1 ± 0.1 b

Analyzing the morphology (Fig. 1) and particle size (Fig. 2, Table 3) of 6 types of powders produced from oat beverage, it can be noticed that some of the applied modifications of the beverage and/or the drying approach caused the significant differences. The concentration (CONC) resulted in decreased PDI, while the application of DASD produced significantly smaller particles. The later phenomenon, caused by decreased drying temperature and decreased stickiness, was presented before for several materials: honey^32^, sour cherry juice concentrate^21^, kiwiberry pulp^19^. The combination of both treatments (CONC-DASD) cumulated two effects, and resulted in similar morphology and particle size as for OAT sample. The addition of oat fiber combined with DASD approach (FIB-DASD) caused the increase of particle size observed in SEM pictures, which can be the result of increased viscosity of feed material (data not shown). However, the difference in the measured particle size between OAT and FIB-DASD was not significant. It was similar to the observation presented by Siccama^33^, who spray dried asparagus concentrate with maltodextrin (MD) and partially replaced MD by asparagus fiber. The replacement of 3% of MD did not affect significantly the particle size and morphology. In the current work, when additionally the concentration step was included (FIB-CONC-DASD), the difference (increase) in particle size was significant, which could be related with the further increase of viscosity affecting the atomization.

#### Yield

Yield of spray drying varied from 16.1 to 59.2%, and was affected significantly both by the type of beverage and the modification applied for oat beverage (Table 3). Majority of values were lower than 50% which is the boarder limit describing the successful spray drying at laboratory and pilot scale according to Bhandari et al^34^. However, it has to be underlined that PBs were dried without the addition of any carrier, which is usually applied for the enhancement of yield. Among four basic PBs the lowest yield was noted for OAT, that is why it was chosen to the next step of work aiming at the improvement of yield by the modification of beverage and/or drying process. Similarly to the formation of particles morphology, it is the relationship between saccharides, their types, and proteins what decides about the drying yield, but also the size of particles can be important as presented below. Low yield for OAT could result from high share of sugars in carbohydrates which together with low share of protein in solids did not allow to produce the dry particles efficiently (sugars depressed glass transition temperature and increased stickiness, while low amount of protein did not help in stickiness reduction on the surface). Moreover, this sample was characterized by the lowest particle size, thus small particles could be lost with outlet air. Such phenomenon of lower yield caused by small particle size was observed before by Samborska et al^35^.–spray drying of gum Arabic (GA) had lower yield than honey + GA even though the stickiness due to sugar content was not present in the case of pure GA drying.

In the second part of the work the oat beverage was spray dried after some initial pretreatments and by modified spray drying at low temperature (DASD) (variants as presented in Table 2). Each of applied modifications caused the improvement of yield, but the most effective was the combination of concentration and drying at low temperature (CONC-DASD). DASD approach have been tested during last five years as a method to reduce the stickiness during spray drying of various materials as honey^32^, white mulberry molasses^36^, kiwiberry pulp^22^. It was effective in the reduction of drying temperature which allows to reduce the necessary carrier addition, or even eliminate it i.e. for blackcurrant concentrate, mango puree, black carrot concentrate^19^. At the same time, it was presented that the decrease of temperature improved the yield i.e. for white mulberry molasses^36^, sour cherry concentrate^21^. In the present work the application of DASD improved the yield up to 44.6% (DASD), while the combination of this drying approach and initial concentration allowed to reach 59.2%. DASD was also combined with additional supplementation of oat beverage with oat fiber (FIB-DASD) and also with initial concentration (FIB-CONC-DASD). Both variants containing fiber had higher yield than basic OAT sample due to lower drying temperature and stickiness. On the contrary, yield was lower than for DASD.

#### Moisture content and water activity

MC in obtained powders varied from 1.3 to 5.2%, while *a*_*w*_ from 0.079 to 0.259 (Table 3). Thus, they met the requirements connected with suitable stability, and minimization of microbial and chemical reactions^37,38^. Padma et al^10^., spray drying rice beverage at inlet air temperature 120–160 °C obtained powders of similar MC in the range 3.26–4.3%, while *a*_*w*_ was higher: 0.250–0.359. Among four basic powdered PBs significantly higher MC was observed in SOY and ALMOND samples. Again, it can be related with the chemical composition of materials—higher share of protein in solids in those samples. Proteins are characterized by high water holding capacity, and moreover the formation of protein-rich surface film acts as a barrier to the diffusion of water molecules during the spray drying leading to an increment of MC^39^. Oat-based powders obtained in the second part of work were significantly different in terms of MC and *a*_*w*_. The lowest values were observed for CONC. It was the sample dried at high temperature, and which contained lower amount of water to be evaporated (because it was removed by preconcentration). The similar sample, but dried at low temperature (CONC-DASD) had significantly higher MC because the driving force for evaporation was lower at lower temperature, even though the humidity of drying air was reduced. Similar effect of higher MC after DASD was presented by Barańska et al^21^. and Samborska et al^36^. The addition of fiber caused the increase of MC and *a*_*w*_ due to increase of particle size and more difficult water diffusion.

### Multivariate analysis of yield and physical properties

#### Yield and physical properties 

**Figure 3:**
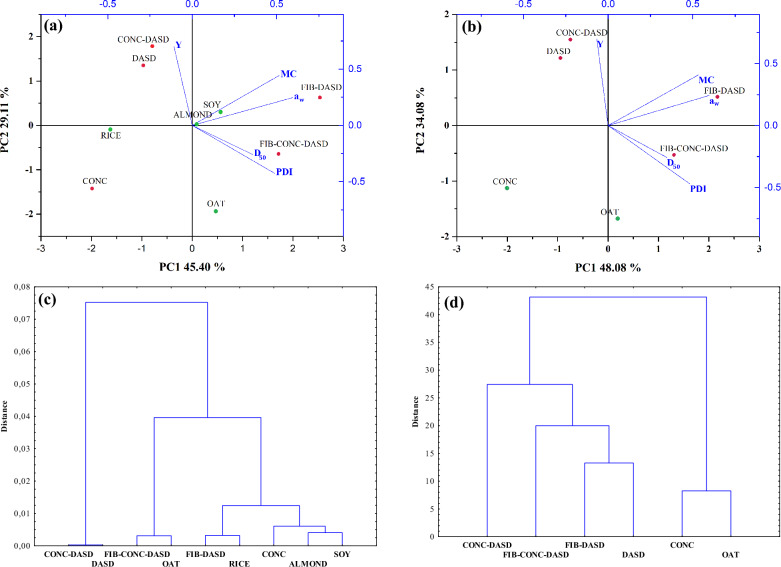
Principal component analysis (PCA) biplot in two-dimensional projection PC1 versus PC2 for PBs (panel **a**) and oat powdered beverages after different treatments (panel **b**); dendrogram trees from HCA for PBs (panel **c**) Pearson correlation and the average linkage criteria were used, and oat powdered beverages (panel **d**) Euclidean distance and the complete-linkage criteria clustering were used.

PCA and HCA were applied to evaluate the relationship amongst the physical parameters such as: water activity (a_w_), moisture content (MC), median particle size (D_50_), polydispersity index (PDI) and the yield of spray drying (Y). The contribution of total variance and eigenvalues for the selected physical properties are presented in Table 4. PCA biplot presented in Fig. 3 panels a–b shows both PC scores and loadings of variables in two dimensional projection, for all PBs (Fig. 3a) and separately for oat samples (Fig. 3b). Moreover, tree dendrograms were also performed for the same samples division as above (Fig. 3 panels c–d). PC1 described the largest possible variation (45.40 and 48.08%), and PC2 the second-highest variation (29.21 and 34.08%) therefore, the following considerations focus only on these components.

**Table 4:** Eigenvalues, percentage of variance, and cumulative percentage in the data used for the PCA calculations obtained from the physical parameters of obtained powders.

Principal component number	Eigenvalue	Percentage of Variance (%)	Cumulative (%)
Plant beverages + oat beverage after different treatments			
1	2.270183	45.40367	45.4037
2	1.460301	29.20602	74.6097
3	0.794743	15.89486	90.5046
Oat beverage after different treatments			
1	2.403937	48.07874	48.0787
2	1.703980	34.07961	82.1583
3	0.779543	15.59086	97.7492

SOY and ALMOND powders were clustered in the centre of biplot, which indicates relatively similar impact of the spray drying process on their physical properties and drying yield. In contrast, RICE and OAT were scattered in the scores plot and did not form any cluster. Analyzing only the oat powders, it can be observed that samples with the addition of fiber (FIB-DASD and FIB-CONC-DASD) were positively correlated with PC1, while samples without this additive were negatively correlated with PC1. The exception is OAT which is located near the border of PC2 axis. Additionally, the influence of the drying temperature (conventional high temperature SD or dehumidified air at low temperature DASD) was clearly visible along PC2 axis, especially when the concentration treatment was applied. It was shown in Fig. 3 (panels a–b) that CONC and CONC-DASD samples are on the opposite sides of PC2. Moreover, DASD and CONC-DASD powders were significantly separated from the rest of the samples and were exclusively characterized by the highest yield of spray drying and the lowest particle size (Table 3). Referring to the results in presented in Table 3 and from the PCA analysis, samples characterized with the highest yield of spray drying process (DASD and CONC-DASD) were spray dried with dehumidified air at low temperature and without addition of oat fiber.

In PCA biplot, correlation between variables (physical properties) is described by the direction, length and angle of the arrows^40^. A low acute angle between two arrow indicate a high positive correlation, while a large obtuse angle indicate a negative one. According to the loading plots presented in the biplot, the most dominant influence along the PC1 axis had MC and *a*_*w*_ parameters (Fig. 3 and Table 5) and along PC2 yield of spray drying. Moreover, MC and *a*_*w*_ show the highest statistically significant positive correlation (Fig. 3 and Table 6). Considering Pearson correlation, other correlations between the examined parameters were much weaker and not statistically significant.

**Table 5:** Correlation coefficients of physical properties with the two major components for all powdered plant beverages (panel a) and oat powdered beverage after different treatments (panel b).

	Coefficients of PC1	Coefficients of PC2
(a)		
Y	− 0.06647	0.69831
a_w_	0.59808	0.24320
MC	0.53258	0.40800
D_50_	0.34791	− 0.25773
PDI	0.48290	− 0.46939
(b)		
Y	− 0.1085	0.70218
a_w_	0.59597	0.24750
MC	0.5163	0.44411
D_50_	0.35748	− 0.25853
PDI	0.48855	− 0.42615

**Table 6:** Pearson correlation on yield of spray drying and physical properties for all powdered plant beverages (panel a) and oat powdered beverage after different treatments (panel b).

	Y	a_w_	MC	D_50_
(a)				
a_w_	0.0304			
MC	0.2083	0.8321*		
D_50_	− 0.1258	0.2133	0.2121	
PDI	− 0.4473	0.5206	0.2181	0.4169
(b)				
a_w_	0.1265			
MC	0.3300	0.9589*		
D_50_	− 0.1112	0.2428	0.1766	
PDI	− 0.6338	0.5236	0.2788	0.5004

*the determined correlation coefficients are statistically significant at the probability level of p < 0.05.

Hierarchical cluster analysis (HCA) was performed on the same data set in order to compare obtained findings from PCA. The results of the HCA analysis are shown through a tree dendrograms in Fig. 3c–d. In the case of all PBs (Fig. 3c), the dendrogram was divided into four main groups (cut-off on 0.01 distance). Similar to PCA, SOY and ALMOND showed similarity and created one cluster, while OAT and RICE were in separate groups. Similarly to the results presented in Fig. 3a, CONC-DASD and DASD samples formed one cluster. On the other hand, for the oat powdered beverages after different treatments (Fig. 3d) two main groups were created (cut off on 30 distance). OAT and CONC samples were clearly separated from other samples, slightly similar to the biplot presented in Fig. 3b. HCA showed that the placement of the PBs on the dendrogram depended on the type of beverages (origin) and additional treatments (concentration to double solids content, addition of oat fiber) or different spray drying approach (see Table 2).

#### FTIR

**Figure 4:**
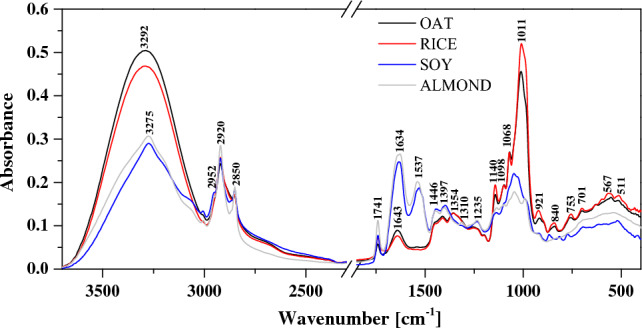
FTIR spectra of four spray dried powdered plant beverages.

**Figure 5:**
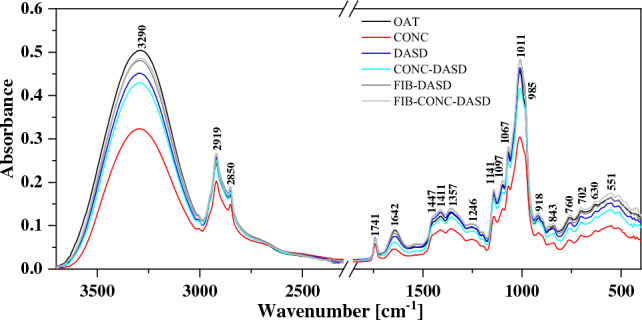
FTIR spectra for different samples of spray dried powdered oat beverage after different treatments (CONC—concentration, FIB—addition of oat fiber, DASD—dehumidified air at low temperature).

PBs were analysed using ATR-FTIR spectroscopy in the midinfrared region (4000–400 cm^−1^). FTIR spectra of four basic powders are presented in Fig. 4, while Fig. 5 presents spectra of oat samples from different treatments. For a more convenient analysis of the obtained FTIR spectra, Table 7 shows the most significant vibrations, together with their assignment to specific functional groups, based on the available literature^13–15,18,41–44^. It is clearly visible that there were noticeable changes in the spectra of four powdered plant beverages reflecting their diverse chemical compositions and the presence of varied class of functional groups like esters, amines, carboxylic acids, aromatics, phenols and amides. On the contrary–spectra of all oat powdered beverages displayed very similar shapes and peaks positions, which differed in intensity across the spectral region.

**Table 7:** The location of the maxima of absorption bands FTIR with arrangement of appropriate vibrations selected for powered plant beverages.

Type and origin of vibrations	Wavenumber (cm^−1^)			
	OAT	RICE	SOY	ALMOND
ν(O–H) and ν(N–H)	3292	3292	3273	3273
ν_as_(–CH_2_) from fatty acid			2954	2953
	2920	2920	2921	2920
ν_s_(–CH_2_) from fatty acid	2850	2850	2850	2850
ν(C = O) from fatty acid	1741	1740	1740	1741
ν(C = O) from amide I, protein	1642	1642	1634	1634
				1626
δ(N–H) from amid II, protein			1543	1538
			1530	
δ(O–CH) and δ(C–C–H)	1449	1449	1447	1447
δ(O–H) in C–OH group + δ(C–H) in the alkenes	1411	1411	1396	1398
δ(C–H) in CH_2_ and CH_3_	1357	1357		
ν(C–N) from amide III, protein	1253	1252	1234	1234
ν(C–H) in carbohydrates or/and ν(C–O) in carbohydrates	1142	1142	1136	1136
ν(C–O) in C–O–C group in carbohydrates	1097	1097	1097	1097
	1069	1069	1075	1073
ν(C–O) in polysaccharides	1011	1009	1023	1026
	986	986	986	990
Carbohydrate ring	917	920	916	916
	894	894	867	867
	840	840	814	827

All spectra showed a broad peak in the –OH stretching region within the range of 3650 to approx. 3000 cm^−1^. For soy and almond spray dried beverages, the –OH stretching region had a triangular shape due to a large contribution from –NH symmetric and asymmetric vibration^45^. The small peak at 3068 cm^−1^ can be attributed to the aromatic C–H stretching band of the amino acid tyrosine^46^. Next, the region of 3000–2800 cm^−1^ is related to the stretching vibration of methylene groups from fatty acid. Vibrations with the maximum at 2920 cm^−1^ related to the asymmetric stretching mode ν_as_(–CH_2_) is accompanied by a smaller, sharp second peak appearing at 2850 cm^−1^ related to the symmetric stretching mode ν_s_(–CH_2_)^15^. Vibrations with the maximum at ~ 1740 cm^−1^ wavenumber can be associated to C=O stretching of ester or carboxylic acid group from the fat presented in the samples.

The largest differences between powdered samples in the IR spectra occurred in the range of the stretching vibration of –OH and –NH group (3600–3000 cm^−1^), amide I and amide II (1700–1500 cm^−1^) and the region of carbohydrates (1200–900 cm^−1^).

Peaks in the range 1700–1200 cm^-1^ correspond to the mixed region containing vibrational bands of fatty acids, proteins and polysaccharides with significant distinction among the spray dried PBs. Between 1700 and 1500 cm^−1^ two major peaks are clearly observed: amide I (νC=O, νC–N) in 1634 cm^−1^ and amide II in 1537 cm^−1^ (δN–H, νC–N), related to peptide bonds. Moreover, a significantly higher absorption values for amide groups (I and II) was observed in the case of powdered samples of SOY and ALMOND compared to the RICE and OAT samples. These peaks are closely linked to the concentration of protein within the samples (Table 1). Bands in the range of 1400–1200 cm^–1^ include information related to the C–O stretching, C–C stretching, C–O–C stretching in lipid and C–N stretching and N–H bending (amide III) in protein^47^. The very important bands within the carbohydrate fingerprint region (1200–900 cm^−1^) can be associated to C−C and C–O stretching modes^13^. Moreover, the a-tocopherol is reported to have a peak at 1086 cm^−1^ related to plane bending of phenyl group^48^. The peaks in the spectral region 1200–900 cm^−1^ for powdered OAT and RICE exhibit strong and characteristic band at 1070, 1010 and 987 cm^−1^ due to high concentration of carbohydrates in comparison to low lipid content (Table 1). The last of the presented region 900–700 cm^−1^ is characteristic of vibrations of C–O in the C–OH group or C–C stretching in the carbohydrate structure.

#### Multivariate analysis of FTIR spectra

**Figure 6:**
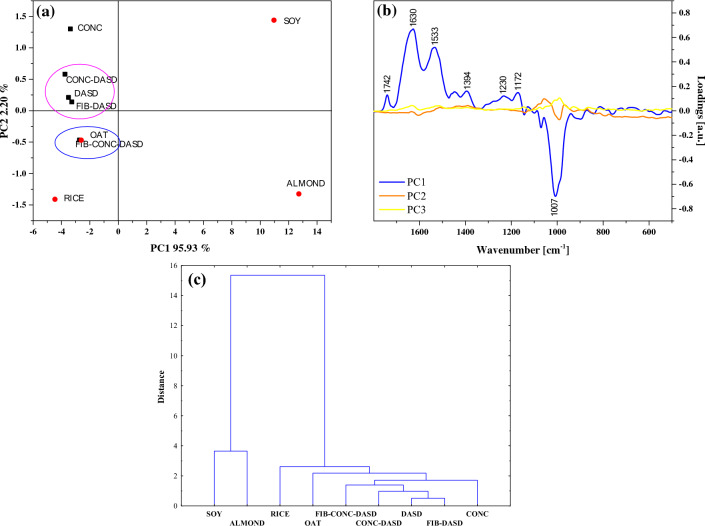
PCA and HCA analysis of the FTIR spectra of PBs and oat beverage after different treatments with mean center as the pre-treatment. The PCA score plot (panel **a**) and loadings (panel **b**). A dendrogram tree (panel **c**) from the HCA. Euclidean distance between the pairs of samples was used as a distance measure and the average linkage criteria was used as an agglomeration method.

**Figure 7:**
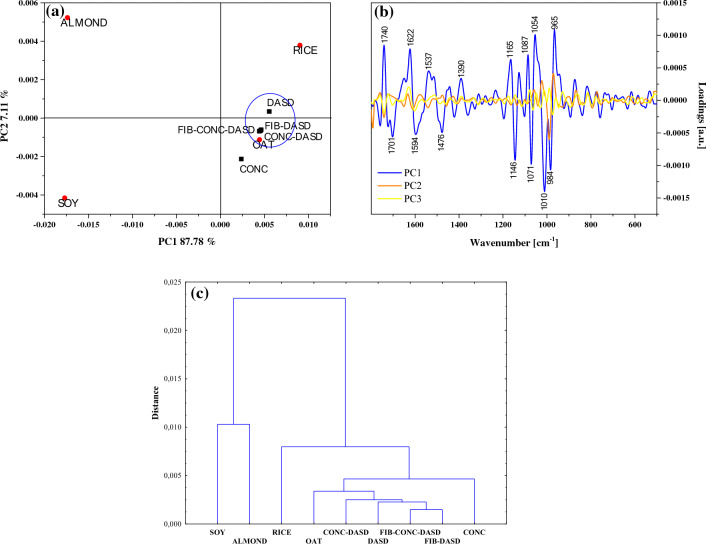
PCA and HCA analysis of the FTIR spectra of PBs and oat beverage after different treatments with mean center and 2nd order derivative as the pre-treatment. The PCA score plot (panel **a**) and loadings (panel **b**). A dendrogram tree (panel **c**) from the HCA. Euclidean distance between the pairs of samples was used as a distance measure and the average linkage criteria was used as an agglomeration method.

**Figure 8:**
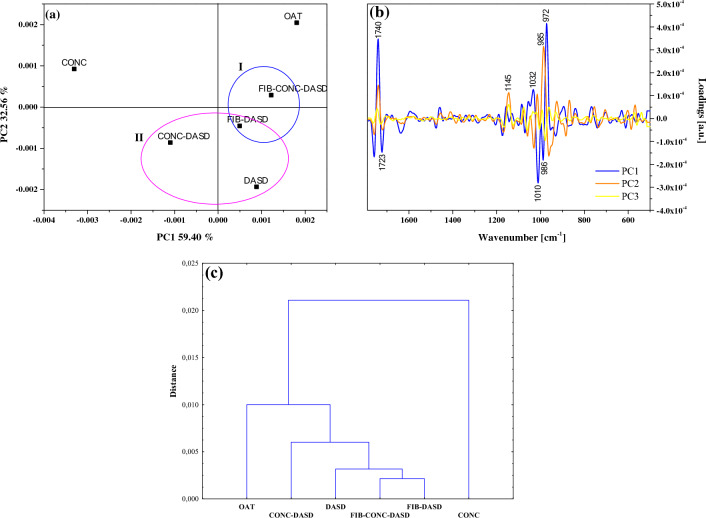
PCA and HCA analysis of the FTIR spectra of oat beverage after different treatments with mean center and 2nd order derivative as the pre-treatment. The PCA score plot (panel **a**) and loadings (panel **b**). A dendrogram tree (panel **c**) from the HCA. Pearson correlation between the pairs of samples was used as a distance measure and the average linkage criteria was used as an agglomeration method.

The combination of the FTIR study with chemometric approaches such as PCA and HCA was employed. IR spectra of food products contain a complex of set data of multiple absorption bands which are strongly overlapped. Therefore, multivariate analysis such as Principal Component Analysis (PCA) and Hierarchical Cluster Analysis (HCA) can be used to process and reduce a huge spectroscopy dataset to perceive the relationship between the variables (e.g. wavenumbers) and be employed in classification studies for a wide variety of food products^49^. This analysis aimed to search patterns between the powdered PBs and to better understand the impact of various approaches in spray drying process. Regarding the evaluation of specific functional groups, interesting differences were identified in the fingerprint region, therefore an exploratory data analysis PCA and HCA was performed between 1800 and 500 cm^−1^ wavenumber. The resulting PCA scores and loadings plots and HCA of the FTIR spectra can be seen in Figs. 6, 7 and 8. First, FTIR data was pre-processed to remove physical effects causing discrimination between samples using mean centering and/or 2nd derivative. The results of this analysis show that different data pre-processing for these samples gives slightly distinct findings.

PCA and HCA was conducted in two ways: (i) to explore differences in powdered RICE, OAT, ALMOND and SOY PBs (Fig. 6—mean center; and 7—mean center + 2nd order derivative), (ii) to investigate the effects of different treatments (concentration, addition of oat fiber, different temperature of spray drying) of oat samples (Fig. 8). The values of eigenvalues and contribution of total variance obtained from principal component analyses of the FTIR spectra are presented in Table 8. In all cases the first two principal components explained over 90%. Therefore, the first two PCs were selected for further analysis. The PCA applied to the pre-processed with mean centering had better results for group clustering than when it was applied additionally 2nd derivative. The first principal component with mean cantering explained 95.93%, while addition of the 2nd derivative explained only 87.78% of the data (all powdered samples).

**Table 8:** Eigenvalues, percentage of variance, and cumulative percentage in the data used for the PCA calculations obtained from FTIR spectra of the plant beverages and oat beverages after different treatments.

Principal component number/spectral pre-processing	Eigenvalue	Percentage of variance (%)	Cumulative (%)
Plant beverages + oat beverages after different treatments			
Mean center			
1	45.57435	95.93275	95.9327
2	1.04352	2.19658	98.1293
3	0.57436	1.20902	99.3383
Mean center + 2nd order derivative			
1	0.00010189	87.78473	87.78473
2	0.00000826	7.11476	94.89949
3	0.00000347	2.98980	97.88929
Oat beverages after different treatments			
Mean center + 2nd order derivative			
1	0.00000358	59.40599	59.4060
2	0.00000196	32.55784	91.9638
3	0.00000023	3.80048	95.7643

Figures 6a and 7a and present a score plot in two-dimensional projection for all powder samples. The first two PCs clearly separate powdered PBs and showed a trend in the separation with significant differences between SOY–ALMOND and RICE–OAT samples, which are located at opposite sides regarding PC1. Additionally, RICE and OAT were located closer to each other on the score plot, which is related to their slight structural similarity, while the ALMOND and SOY samples are definitely separated from the rest. According to the loadings plots (Fig. 6b and 7b) PC1 mainly describe the variability within the fingerprint region regardless of the used pre-processing methods. PC1 variance was most influenced by amide I, II (1700–1500 cm^−1^) and carbohydrate (1100–900 cm^−1^) regions which is contribute to the diversity among PBs. PCA obtained from FTIR spectra confirmed that the selected plant beverages differed in their chemical composition (Fig. 6a and 7a), while in this case the physical properties, SOY and ALMOND were slightly similar (Fig. 3a).

Since the type of PBs had a relevant impact into PCA, oat beverages subjected to different treatments were more difficult to distinguish on the scatter plots of PC1 versus PC2. In this case oat samples spray dried at low drying temperature with dehumidified air application (DASD) created separate group, while CONC (concentrated 2×, spray-dried conventionally) was significantly distant from the cluster.

Additionally, for a more in-depth analysis of the impact of the used additives (oat fiber) and the spray drying approach, the PCA analysis was performed for oat samples (Fig. 8). From the score plot (Fig. 8a), it can be seen that the addition of oat fiber and the used temperature of spray drying were factors determining the division and clustering into groups. It can be observed that two main groups were created: I—samples with oat fiber (FIB-DASD and FIB-CONC-DASD), II—samples which were concentrated and spray dried at lower temperature. The influence of applied temperature can be clearly observed through distinction of OAT and CONC (spray dried conventionally) from DASD, CONC-DASD, FIB-DASD and FIB-CONC-DASD (low drying temperature with dehumidified air application). The PCA loadings plot indicates that maximum contribution to spectral differences is due to changes in the vibrations associated to C=O stretching from the fatty acid (1750–1720 cm^−1^) and C–O, C–O–C stretching modes (1100–900 cm^−1^) from carbohydrate. The arrangements of points on the score plot (Fig. 8a) is related to the differences in the intensities for the abovementioned spectral regions.

Hierarchical cluster analysis (HCA) was conducted on the same data set between 1800 and 500 cm^−1^ wavenumber, and the results of the HCA analysis are shown through a dendrograms in Figs. 6c and 8c. It can be observed, a very comparable grouping between HCA and PCA analysis. The dendrograms obtained using average linkage method and Euclidean distances (Fig. 6c and 7c) are divided into two main clusters, both with two subclusters. The first group contains SOY and ALMOND powders, and the second one contains RICE and OAT samples after different spray drying approaches. In Fig. 8c, the resulting dendrogram grouped oat powdered samples into two main clusters. CONC (concentrated 2×) was clearly separated from other samples, similar to results presented in the score plot (Fig. 8a). The FTIR spectra of oat samples after different treatments displayed very similar shapes and peaks in the fingerprint region, but sample CONC was the most differed in intensity.

## Conclusions 

The study shown that it is possible to produce the powdered plant-based beverages without the additional substances—carriers by conventional spray drying at high temperature. However, as the yield for four basic beverages was lowest was noted for oat beverage, this sample was choosen for further works to improve the yield. The modifications which were applied were effective—the yield was significantly improved (the highest was achieved for the concentrated beverage dried using DASD). The physical properties of powders were typical for spray dried materials.

Multivariate analyses such as PCA and HCA were applied to study the relationship between the physical properties of spray dryied plant beverages. It was shown that the SOY and ALMOND samples formed one cluster, while the RICE and OAT samples differed significantly from the others. Moisture content and water activity had the highest contribution to separating samples into the PC1 axis. Moreover, the arrangement of the oat samples in the score plot was mostly affected by the applied drying approach and the addition of fibre.

The use of FTIR spectral analysis revealed noticeable differences in the intensity of bands characteristic for amide I and II from proteins and carbohydrates present in plant beverages. The spectral differences in the position and intensity of the bands were mainly related to the different mass ratios of macronutrients present in samples. Moreover, examined whether a combination of FTIR spectroscopy and subsequent chemometric data analysis (PCA and HCA) could be applied to differentiate plant beverages of different origin as well as various modifications during spray drying. The PCA loadings plot indicated that the greatest impact on the grouping of samples had spectral region attributed to stretching vibration of C=O from the fatty acid (1750–1720 cm^−1^), amide I and II (1700–1500 cm^−1^) and C–O, C–O–C stretching modes (1100–900 cm^−1^) from carbohydrate. Powdered plant beverages showed no similarity, while samples differing in the preparation method formed one cluster, indicating a minor impact of the different spray drying methods on FTIR spectra.

## Data Availability

The datasets used and/or analysed during the current study available from the corresponding author on reasonable request.
